# Distinguishing vulnerable clients from psychotic patients with follow-up mortality data

**DOI:** 10.1192/bjo.2021.90

**Published:** 2021-06-18

**Authors:** Aggrey Burke

**Affiliations:** Cope mental health foundation

## Abstract

**Aims:**

The aim of the present study is to determine whether vulnerable non-psychotic clients presenting in court proceedings do not share the same mortality profile as psychotic patients in similar environments. It is hypothesised that the two display quite separate mortality profiles.

**Background:**

The increased mortality of psychiatric patients and prisoners has been documented but less is known of the outcomes among other vulnerable populations .

The population for study is a consecutive series of assessments in court proceedings of carers of children at risk and violent offenders.

**Method:**

Assistants not involved in the initial assessments transferred data from case notes and this material was transferred to computer files. Statistical analysis SPSS19

Formal psychiatric diagnoses were those agreed in court proceedings. National mortality records were searched and copies of death certificates obtained. A small number of cases known to have returned overseas were excluded. 772 cases were studied. One in five were assessed in prison, twice as many gave a history of violent criminal behaviour. Over a half suffered abuse or neglect or admitted to being unhappy in childhood. Three subgroups have been identified: Vulnerable with no psychotic illness(60%), psychosis with no evidence of personality disorder or of mixed psychosis(18%), linked psychosis(22%). It was found that demographic variables , deprivation factors, adverse childhood experiences and outcomes and clinical variables are in excess among linked psychotics compared with other groups. Linear regression of unnatural death among psychotic patients identifies five risk factors. The distribution of high-risk factors among linked psychosis is more than twice that found in other groups.

**Result:**

Natural mortality is most evident among clients suffering from psychosis without personality disorder or mixed disorder.Unnatural mortality is more than 10 times greater among patients with linked psychosis, compared with those with no psychosis and four times greater than other psychoses. Risk factors for unnatural mortality are: physical illness, stressful relationship, violence to self or others, detained and history of behaviour disorder.

**Conclusion:**

The findings of the present study demonstrate that vulnerable clients without psychosis are less likely to die by unnatural causes than clients who suffer psychosis coexisting with personality disorder or mixed psychosis. The null hypothesis is upheld. The findings suggest that risk assessment of vulnerable populations should take account of risk factors of unnatural death which have been identified in this study.

